# Rezvilutamide combined with ADT in an HIV-positive patient with high-volume mHSPC on efavirenz-based antiretroviral therapy: a case report

**DOI:** 10.3389/fonc.2026.1864715

**Published:** 2026-08-10

**Authors:** Heng Zhang, Tailin He, Zhiyuan Li, Yujie Gao, Yan Li, Yundie Zhou, Haifeng Wang

**Affiliations:** Department of Urology, Ya’an People’s Hospital, Ya’an, China

**Keywords:** androgen receptor signaling inhibitors, antiretroviral therapy, case report, HIV, metastatic hormone-sensitive prostate cancer (mHSPC), rezvilutamide, treatment intensification

## Abstract

This case report describes a patient with metastatic hormone-sensitive prostate cancer (mHSPC), high tumor burden, and concurrent HIV infection receiving continuous efavirenz (a potent CYP3A4 inducer), who was treated with an intensified regimen based on androgen deprivation therapy (ADT) combined with the novel androgen receptor inhibitor rezvilutamide. Under rigorous multidisciplinary monitoring, this regimen achieved a rapid and profound response, characterized by a decline in prostate-specific antigen (PSA) levels from 21,788.320 ng/mL to 4.315 ng/mL, significant clinical improvement, sustained HIV viral suppression, and a favorable short-term tolerability profile. This case directly addresses the controversies and challenges regarding the use of intensified therapy in patients with comorbidities in current clinical practice, suggesting that key drug-drug interaction barriers may be manageable through personalized strategies and proactive management. This case provides a practical reference for implementing guideline-recommended treatment intensification principles in similar complex clinical scenarios, offering clinically relevant insights.

## Introduction

Metastatic hormone-sensitive prostate cancer (mHSPC) represents a critical stage in the progression of prostate cancer, in which the standard of care has evolved from androgen deprivation therapy (ADT) alone to ADT combined with novel endocrine therapies, significantly improving patient prognosis (1). However, with advances in antiretroviral therapy, the life expectancy of individuals living with HIV has substantially increased, leading to a marked rise in the incidence of non-AIDS-defining malignancies, including prostate cancer, within this population (2). Patients with mHSPC and concurrent HIV infection constitute a unique clinical subgroup whose management faces dual challenges: on one hand, applying effective antineoplastic regimens to control aggressive disease (3, 4), and on the other, appropriately managing complex interactions between antineoplastic and antiviral agents (5–7). An integrated, multidisciplinary approach is essential for optimizing outcomes in such patients with concurrent malignancies and chronic infections (8).

The present case is notable because the patient was diagnosed with high-volume mHSPC concurrent with well-controlled HIV infection, with an antiretroviral regimen containing efavirenz, a potent CYP3A4 enzyme inducer (5). Novel androgen receptor inhibitors (ARi), including rezvilutamide (9), are currently among the core agents for intensified treatment of mHSPC. However, as substrates of CYP3A4, their concomitant use with inducers such as efavirenz raises a theoretical concern of reduced plasma concentrations and diminished efficacy due to accelerated drug metabolism (10, 11). To date, clinical data regarding the use of rezvilutamide in HIV-positive patients are extremely scarce, and direct evidence on efficacy and safety in this context remains lacking.

This case report provides a detailed account of the treatment course and preliminary outcomes of ADT combined with rezvilutamide (alongside an efavirenz-based antiretroviral regimen) in a patient with high-volume mHSPC and concurrent HIV infection. Under close multidisciplinary monitoring, this combination regimen achieved rapid and deep PSA response and significant symptom improvement while maintaining sustained suppression of HIV viral load, without severe adverse events or immune reconstitution inflammatory syndrome. This experience suggests that personalized intensified therapy, under adequate monitoring and support, may be feasible in this specific patient subset, contributing to the limited evidence base in this area.

## Case description

A 68-year-old male presented with recurrent lower back pain for six months, accompanied by voiding difficulties. The symptoms began six months prior; initially, the patient disregarded the symptoms and self-administered oral analgesics, with no significant relief. Approximately one month before presentation, the patient experienced progressively worsening lower back pain, prompting a visit to a local hospital, where testing revealed a PSA level >100 ng/mL. For further diagnosis and treatment, the patient was transferred to our hospital. The patient has a four-year history of HIV infection and has been regularly receiving antiretroviral therapy with oral lamivudine, efavirenz, and tenofovir. Over ten years ago, he underwent internal fixation surgery for a tibial fracture in the left lower limb, with the hardware remaining *in situ*.

**Figure 1:**
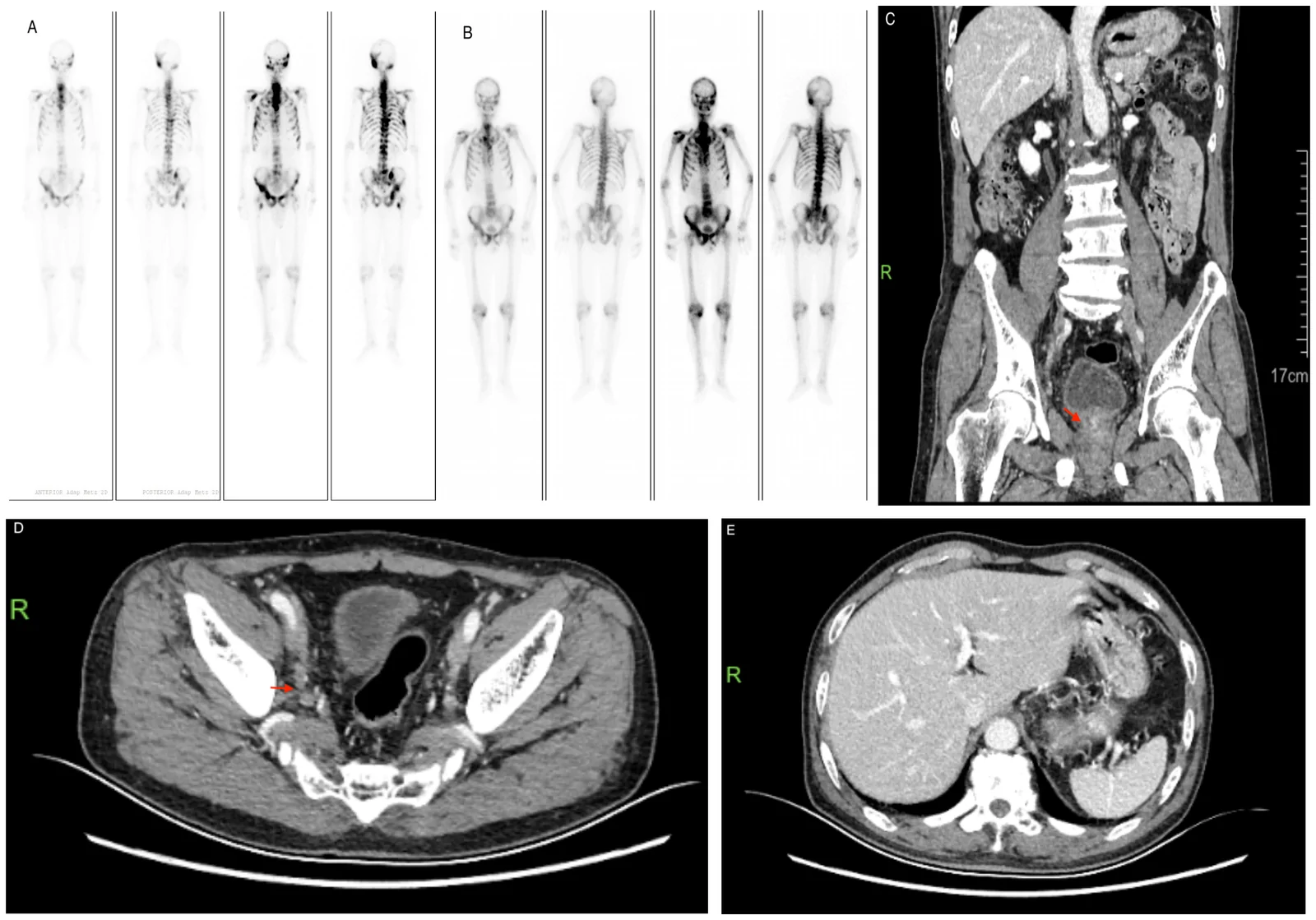
**(A)** Pre-treatment ECT (2025.9.23): Extensive areas of increased radiotracer uptake throughout the axial and appendicular skeleton, consistent with multifocal osteoblastic bone metastases. **(B)** Post-treatment ECT (2026.4.21): Decreased intensity and slight reduction in the extent of bone metastatic lesions compared with prior imaging. **(C–E)** Contrast-enhanced abdominal CT (2026.4.23): **(C)** Irregular prostatic morphology with indistinct boundaries relative to the rectum. **(D)** Pelvic lymph nodes with indistinct margins. **(E)** Liver with no evidence of metastatic lesions.

Physical examination after admission: Digital rectal examination revealed significant prostate enlargement with multiple palpable hard nodules. Marked tenderness was noted in major joints throughout the body and the lumbosacral region. The Eastern Cooperative Oncology Group (ECOG) performance status score was 1. Laboratory tests showed a hemoglobin level of 99 g/L (mild anemia); hepatic and renal function and coagulation parameters were within normal limits. Serum PSA levels were markedly elevated at 21,788.320 ng/mL. Pre-admission evaluations confirmed well-controlled HIV infection, evidenced by a CD4+ T-cell count of 519 cells/μL, a CD8+ T-cell count of 322 cells/μL, and an HIV viral load below the detection limit. Bone scintigraphy (ECT) revealed focal areas of increased radiotracer uptake in the skull, sternum, multiple vertebral bodies, ribs, pelvis, bilateral scapulae, proximal humeri, proximal femurs, and the left tibia, consistent with extensive multifocal bone metastases (**Figure 1A**).

**Figure 2:**
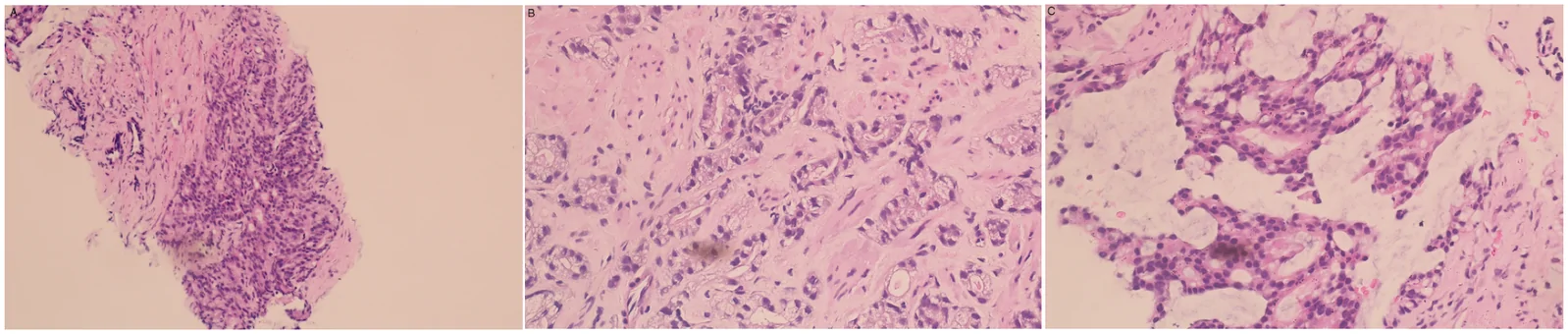
Histopathology of prostatic adenocarcinoma (H&E stain, 2024.9.26). **(A)** Low-power field showing invasive glandular structures with stromal connective tissue hyperplasia. **(B, C)** High-power magnification revealing malignant glands with marked nuclear atypia, prominent nucleoli, and absence of basal cells.

To characterize the primary tumor, the patient underwent transperineal systematic prostate biopsy under ultrasound guidance with local anesthesia. Pathological examination revealed prostatic acinar adenocarcinoma (12 cores obtained, 10 positive for carcinoma; tumor involvement ranged from 30% to 100%), Gleason score 4 + 4 = 8 (WHO/ISUP Grade Group 4). Immunohistochemical analysis demonstrated approximately 25% Ki-67 positivity in tumor cells. The cells exhibited positive expression of EGFR, P504S, PSA, and the androgen receptor, while lacking expression of P63 and CK34βE12 (**Figures 2A–C**). The final clinical diagnosis was high-volume mHSPC with controlled HIV infection.

A comprehensive treatment plan was developed, tailored to the patient’s presentation. The endocrine therapy regimen comprised combined treatment with the gonadotropin-releasing hormone agonist goserelin (10.8 mg subcutaneously every 12 weeks) and the novel androgen receptor inhibitor rezvilutamide (240 mg orally once daily). To manage bone metastases and prevent skeletal-related events, denosumab (120 mg subcutaneously once monthly) was administered, concomitant with daily vitamin D3 supplementation (800 IU). During the first month of treatment, safety and efficacy were closely monitored; liver and kidney functions were assessed biweekly (subsequently adjusted to every 1–3 months), while serum PSA and testosterone levels were monitored monthly at each follow-up visit. The patient continued the existing antiretroviral regimen (lamivudine, efavirenz, tenofovir) with close monitoring of HIV viral load and immune function. Due to the presence of retained metal internal fixation devices in the left lower limb, which contraindicate MRI examination, only contrast-enhanced CT of the abdomen and pelvis was performed for response evaluation. Both baseline and follow-up CT scans were performed using an identical contrast-enhanced protocol at our institution, ensuring comparability of assessment.

The patient tolerated the treatment regimen well. Adverse events (AEs) were systematically assessed and graded according to the Common Terminology Criteria for Adverse Events (CTCAE) version 5.0 at each visit. No treatment-related AEs of any grade were observed, including fatigue, hypertension, falls, cardiovascular events, or neurocognitive symptoms. Hypocalcemia and osteonecrosis of the jaw (associated with denosumab) were also not observed. Pain resolved rapidly within one month of treatment initiation, concurrent with improvements in quality of life; the ECOG performance status improved from 1 at baseline to 0 at the 2-month follow-up and remained at 0 thereafter. The total FACT-P (Functional Assessment of Cancer Therapy–Prostate, version 4) score increased from a baseline of 62 to 124 at the 7-month follow-up. The FACT-P total score ranges from 0 to 156, with higher scores indicating better health-related quality of life. The observed improvement of 62 points substantially exceeds the established minimal clinically important difference (MCID) of ≥10 points for the FACT-P total score, as consistently used in mHSPC clinical trials (1, 12), indicating clinically meaningful improvement. Notable improvements were observed in the Prostate Cancer Subscale (PCS) and Physical Well-Being Subscale (PWB), reflecting significant alleviation of bone pain and urinary symptoms (**Table 1**).

**Table 1:** Clinical timeline and treatment response.

Timeline	Clinical event/intervention	Key findings	Notes
Baseline (2025.9.23)	Diagnosis confirmed	ECOG: 1 PSA: 21,788.320 ng/mL ECT: Multiple osteoblastic metastases FACT-P: 62	Documented baseline tumor burden
Initial treatment (2025.9.29)	Goserelin 10.8 mg q12w + Rezvilutamide 240 mg qd + Denosumab 120 mg qm + Vitamin D3–800 IU qd	ECOG: 1	Initiation of combination therapy alongside ART
~2 weeks (2025.10.13)	Review of liver/kidney function	ECOG: 1 FACT-P: 87	Normal liver/kidney function, no AEs
~1 month (2025.10.27)	Routine follow-up	ECOG: 1 FACT-P: 98 PSA: 1,027.687 ng/mL	Rapid PSA response (>95% reduction)
~2 months (2025.11.28)	Routine follow-up	ECOG: 0 FACT-P: 112 PSA: 32.238 ng/mL	Complete resolution of bone pain
~3 months (2025.12.30)	Routine follow-up + HIV monitoring	ECOG: 0 FACT-P: 107 PSA: 20.681 ng/mL	HIV VL suppressed; no IRIS
~4 months (2026.1.29)	Routine follow-up	ECOG: 0 FACT-P: 117 PSA: 13.664 ng/mL	Sustained treatment response
~5 months (2026.2.24)	Routine follow-up	ECOG: 0 FACT-P: 121 PSA: 9.028 ng/mL	Stable long-term efficacy
~6 months (2026.3.22)	Routine follow-up + HIV monitoring	ECOG: 0 FACT-P: 125 PSA: 6.145 ng/mL	Deep PSA response; HIV VL suppressed
~7 months (2026.4.21)	Response evaluation ECT + CT	ECOG: 0 FACT-P: 124 PSA: 4.315 ng/mL ECT: Partial response CT: No visceral metastasis	Deep PSA remission; imaging confirms tumor control

ECT, emission computed tomography; CT, computed tomography;ECOG, Eastern Cooperative Oncology Group; PSA, prostate-specific antigen; FACT-P, Functional Assessment of Cancer Therapy–Prostate (version 4); ART, antiretroviral therapy; AE, adverse event; VL, viral load; IRIS, immune reconstitution inflammatory syndrome; q12w, every 12 weeks; qd, once daily; qm, once monthly.

Serum PSA levels exhibited a rapid and substantial decline: to 1,027.687 ng/mL after the first month of treatment (a reduction >95%) and further to 4.315 ng/mL by the seventh month (>99.98% reduction). Serum testosterone levels decreased from a baseline of 4.27 ng/mL to 0.14 ng/mL at 1 month and remained in the castration range (<0.5 ng/mL, per current prostate cancer guidelines) throughout the follow-up period, confirming adequate testosterone suppression (**Table 2**). ECT performed at 7 months post-treatment showed reduced radiotracer uptake intensity and a decrease in the extent of pre-existing bone metastases, with no new metastatic lesions detected, suggesting effective control of tumor burden (**Figure 1B**). Contrast-enhanced CT showed no evidence of visceral metastases (**Figures 1C–E**). Regarding HIV, the viral load remained consistently below the limit of detection, CD4+ and CD8+ T-lymphocyte counts remained stable, and no clinical manifestations suggestive of immune reconstitution inflammatory syndrome were observed (**Table 2**).

**Table 2:** Longitudinal laboratory and HIV-related follow-up data.

Variable	Baseline (2025.9.23)	~1 month (2025.10.27)	~2 months (2025.11.28)	~3 months (2025.12.30)	~4 months (2026.1.29)	~5 months (2026.2.24)	~6 months (2026.3.22)	~7 months (2026.4.21)	Unit	Reference range
PSA	21,788.320	1,027.687	32.238	20.681	13.664	9.028	6.145	4.315	ng/mL	0–4
Testosterone	4.27	0.14	0.11	0.13	0.13	0.15	0.14	0.19	ng/mL	1.23–8.13
Hb	99	104	—	116	—	—	125	—	g/L	130–175
PLT	307	328	—	359	—	—	342	—	×10^9^/L	125–350
ALT	9.4	12.4	—	13.4	—	—	15.6	—	U/L	9–50
AST	26.0	31.3	—	30.9	—	—	26.9	—	U/L	40–130
ALB	37.4	39.7	—	40.2	—	—	41.0	—	g/L	40–55
CREA	90	91	—	89	—	—	85	—	µmol/L	59–104
BUN	6.04	6.59	—	5.28	—	—	4.32	—	mmol/L	3.1–9.5
GFR	73.77	74.01	—	76.83	—	—	79.05	—	mL/min	>80
UA	336	340	—	229	—	—	136	—	µmol/L	202.3–416.5
ALP	639.5	350	—	208.6	—	—	164.2	—	U/L	40–130
**CD4+ Count**	**519**	**—**	**—**	**732**	**—**	**—**	**639**	**—**	**cells/µL**	**400–1610**
**CD8+ Count**	**322**	**—**	**—**	**465**	**—**	**—**	**395**	**—**	**cells/µL**	**150–1250**
**CD4+/CD8+ Ratio**	**1.61**	**—**	**—**	**1.57**	**—**	**—**	**1.62**	**—**		**1.0–2.5**
**HIV Viral Load**	**Undetected**	**—**	**—**	**Undetected**	**—**	**—**	**Undetected**	**—**	**copies/mL**	**—**

Key HIV-related parameters (CD4+ count, CD8+ count, CD4+/CD8+ ratio, HIV viral load) are shown in bold. Throughout the follow-up period, HIV viral load remained undetectable, and CD4+ T-cell counts remained within the normal range, indicating sustained HIV control.

Laboratory parameters marked with “—” were not measured at that time point per the follow-up protocol. PSA, prostate-specific antigen; Hb, hemoglobin; PLT, platelets; ALT, alanine aminotransferase; AST, aspartate aminotransferase; ALB, albumin; CREA, creatinine; BUN, blood urea nitrogen; GFR, glomerular filtration rate; UA, uric acid; ALP, alkaline phosphatase.

## Discussion

With the widespread use of antiretroviral therapy, the life expectancy of individuals living with HIV has increased significantly, making non-AIDS-defining malignancies such as prostate cancer an increasingly important clinical issue in this population (13, 14). However, data on the management of mHSPC in patients with coexisting HIV remain limited. Retrospective analyses have demonstrated that prognosis in HIV-positive prostate cancer patients is not significantly associated with HIV status, and treatment tolerability appears favorable (4). Nevertheless, these studies primarily included patients receiving traditional ADT or earlier endocrine agents. The standard treatment for mHSPC in the general population now comprises ADT combined with novel endocrine therapies such as abiraterone, enzalutamide, darolutamide, or apalutamide, a strategy that significantly prolongs survival (1, 15). Data on the use of novel ARi in mHSPC patients receiving ART containing potent CYP3A4 inducers such as efavirenz remain extremely scarce. A recent comprehensive review of novel hormone therapies, including rezvilutamide, emphasized the importance of understanding resistance mechanisms and long-term surveillance for therapeutic resistance in advanced prostate cancer (7).

To our knowledge, this may represent one of the first documented cases of successful treatment with ADT combined with rezvilutamide in an HIV-infected patient continuously receiving the potent CYP3A4 inducer efavirenz. A systematic literature search was conducted in PubMed, Embase, and Web of Science Core Collection using combinations of the terms “rezvilutamide” OR “SHR3680”; “HIV” OR “HIV-positive”; “prostate cancer” OR “prostatic neoplasms” OR “mHSPC”; and “efavirenz” OR “CYP3A4 inducer”. No language or date restrictions were applied. Across the three databases, PubMed and Web of Science yielded zero results. Embase retrieved one record, which after full-text screening was determined to be unrelated (**Supplementary Table 1**). No prior clinical reports of rezvilutamide use in HIV-positive prostate cancer patients receiving efavirenz-based ART were identified. In this case, despite ongoing efavirenz treatment, standard-dose rezvilutamide combined with ADT achieved a profound response, with PSA declining from a markedly elevated baseline of 21,788.320 ng/mL to 4.315 ng/mL. Imaging assessment of bone metastases showed regression of existing lesions without new lesions.

A central question raised by this case concerns the management of drug-drug interactions. Efavirenz, as a potent CYP3A4 inducer, would be expected to accelerate the metabolism of rezvilutamide, a CYP3A4 substrate, potentially reducing its plasma concentration and efficacy (5, 10, 11). The favorable clinical response in this patient may suggest that rezvilutamide possesses a relatively wide therapeutic window, or that interindividual pharmacokinetic variability allowed sufficient drug exposure despite the presence of an inducer. However, several important limitations must be acknowledged: no pharmacokinetic measurements or plasma concentrations of rezvilutamide or its metabolites were assessed, and no therapeutic drug monitoring was performed. Therefore, it remains unclear whether efavirenz had a minimal effect on rezvilutamide exposure or whether drug exposure was reduced but remained therapeutically sufficient. Furthermore, the observed response resulted from combined ADT and rezvilutamide and cannot be attributed to rezvilutamide alone. These observations are hypothesis-generating rather than definitive, and the findings should be interpreted cautiously. The case suggests that in selected patients under close monitoring, such drug interactions may not preclude clinical benefit, but this single clinical observation requires further pharmacokinetic data and prospective validation before broader clinical recommendations can be made.

Regarding the risk of immune reconstitution inflammatory syndrome (IRIS), the patient had received stable ART for four years, with an undetectable baseline HIV viral load and preserved CD4 count, which differs from the typical setting where IRIS occurs after ART initiation or intensification. IRIS was not observed in this case, consistent with the patient’s well-controlled HIV status and the relatively modest immunomodulatory effects of ARi compared with immunotherapies.

Several additional limitations warrant consideration. First, as a single case report, the findings have limited generalizability and cannot be extrapolated to all mHSPC patients with comorbid HIV infection. Second, the imaging response assessment was based primarily on bone scintigraphy, which has well-recognized limitations in evaluating osteoblastic prostate cancer metastases, as treatment-related bone healing may produce similar findings. Standardized imaging response criteria (e.g., RECIST 1.1 for soft tissue and PCWG3 for bone) were used as guidance, but the predominantly osteoblastic nature of the metastases limits the precision of response quantification on bone scan alone. Third, the follow-up period of 7 months is relatively short; long-term progression-free survival, overall survival, and late toxicities or delayed adverse events cannot yet be assessed. The conclusion regarding tolerability should therefore be interpreted as favorable short-term tolerability. Fourth, the absence of pharmacokinetic data means that conclusions regarding the drug-drug interaction remain theoretical. Future prospective studies with pharmacokinetic evaluation, therapeutic drug monitoring, and longer follow-up are needed. Additionally, emerging liquid biopsy approaches may offer non-invasive tools for monitoring treatment response and disease progression in such complex clinical scenarios (16).

In summary, this case suggests that for patients with high-tumor-burden mHSPC and controlled HIV who are receiving a potent CYP3A4 inducer such as efavirenz, intensified therapy including rezvilutamide may be feasible under conditions of prudent risk assessment, multidisciplinary collaboration, and rigorous clinical monitoring. The case highlights key principles — assess risks, make individualized decisions, strengthen monitoring, and foster team collaboration — that may serve as a practical reference for addressing the common challenge of concurrent malignancy and chronic infection. An integrated, multidisciplinary, and patient-centered management strategy is essential for optimizing outcomes in such complex scenarios (8). These findings should be considered exploratory and hypothesis-generating, and further research is warranted to establish the safety and efficacy of this approach in larger populations.

## Patient perspective

Prior to treatment initiation, the patient experienced severe systemic bone pain that significantly impacted daily activities and sleep. Following the commencement of combined rezvilutamide and ADT therapy, while continuing efavirenz-based antiretroviral treatment, the patient reported significant pain relief within one month. Throughout the treatment course, no drug-related adverse events were reported by the patient. The patient noted marked improvements in appetite, energy levels, urinary symptoms, and sleep quality. He expressed sincere gratitude to the medical team for their timely diagnosis and effective management. The patient demonstrated excellent treatment adherence and strictly followed the follow-up schedule.

## Data Availability

The original contributions presented in the study are included in the article/**Supplementary Material**. Further inquiries can be directed to the corresponding author.

## References

[1] Wang L Paller CJ Hong H De Felice A Alexander GC Brawley O . Comparison of systemic treatments for metastatic castration-sensitive prostate cancer: a systematic review and network meta-analysis. JAMA Oncol. (2021) 7:412–20. doi: 10.1001/jamaoncol.2020.6973 33443584 PMC7809610

[2] Shiels MS Pfeiffer RM Gail MH Hall HI Li J Chaturvedi AK . Cancer burden in the HIV-infected population in the United States. J Natl Cancer Inst. (2011) 103:753–62. doi: 10.1093/jnci/djr076 21483021 PMC3086877

[3] Wosnitzer MS Lowe FC . Management of prostate cancer in HIV-positive patients. Nat Rev Urol. (2010) 7:348–57. doi: 10.1038/nrurol.2010.61 20421876

[4] Markowski MC Pirsl F Keruly JC Chander G Moore RD Lau B . Clinical management and outcomes of HIV-positive patients newly diagnosed with prostate cancer: a single institution experience. Prostate Cancer Prostatic Dis. (2024) 27:144–6. doi: 10.1038/s41391-022-00586-7 36057651 PMC9981812

[5] Kharasch ED Whittington D Ensign D Hoffer C Bedynek PS Campbell S . Mechanism of efavirenz influence on methadone pharmacokinetics and pharmacodynamics. Clin Pharmacol Ther. (2012) 91:673–84. doi: 10.1038/clpt.2011.276 22398970 PMC3600645

[6] Londero A Fusi M Cinausero M Tascini C Gervasoni C Cattaneo D . Effects of the androgen receptor inhibitor enzalutamide on the pharmacokinetics of dolutegravir and tenofovir: a case report. Aids. (2022) 36:1603–5. doi: 10.1097/qad.0000000000003269 35979833

[7] Wang Z Wang J Li D Wu R Huang J Ye L . Novel hormone therapies for advanced prostate cancer: Understanding and countering drug resistance. J Pharm Anal. (2025) 15(9):101232. doi: 10.1016/j.jpha.2025.101232 41079787 PMC12512997

[8] Sun P Wan Z Liu Y . Towards comprehensive chronic disease control: prevention, early detection, and integrated management. Med Bull. (2026) 2:20–33. doi: 10.1002/mdb2.70020 41531421

[9] Gu W Han W Luo H Zhou F He D Ma L . Rezvilutamide versus bicalutamide in combination with androgen-deprivation therapy in patients with high-volume, metastatic, hormone-sensitive prostate cancer (CHART): a randomised, open-label, phase 3 trial. Lancet Oncol. (2022) 23:1249–60. doi: 10.1016/s1470-2045(22)00507-1 36075260

[10] Zhu D Huang H Ye S Wang Y Li S Li K Wu J . Evaluation of the pharmacokinetic interactions of rezvilutamide (SHR3680) with midazolam, S-warfarin and omeprazole in patients with prostate cancer. (2022). doi: 10.22541/au.167117778.83793750/v1

[11] Dai T Xu Z Huang L Wang Y Zeng G Ye M . A single-centre, open-label, single-arm, fixed-sequence pharmacokinetic study of SHR3680 on repaglinide and bupropion in prostate cancer patients. Br J Clin Pharmacol. (2023) 89:874–86. doi: 10.1111/bcp.15528 36098470 PMC10092624

[12] Cella D Nichol MB Eton D Nelson JB Mulani P . Estimating clinically meaningful changes for the Functional Assessment of Cancer Therapy--Prostate: results from a clinical trial of patients with metastatic hormone-refractory prostate cancer. Value Health. (2009) 12:124–9. doi: 10.1111/j.1524-4733.2008.00409.x 18647260

[13] Pantanowitz L Bohac G Cooley TP Aboulafia D Dezube BJ . Human immunodeficiency virus-associated prostate cancer: clinicopathological findings and outcome in a multi-institutional study. BJU Int. (2008) 101:1519–23. doi: 10.1111/j.1464-410x.2008.07474.x 18384640

[14] Ong WL Manohar P Millar J Royce P . Clinicopathological characteristics and management of prostate cancer in the human immunodeficiency virus (HIV)-positive population: experience in an Australian major HIV centre. BJU Int. (2015) 116:5–10. doi: 10.1111/bju.13097 26315395

[15] Flauto F Neola G Caso C Signori A Fornarini G Scagliarini S . Evaluating treatment efficacy in metastatic hormone-sensitive prostate cancer patients with visceral disease: a systematic review and network meta-analysis. Eur Urol Oncol. (2026) 9:447–58. doi: 10.1016/j.euo.2025.06.008 40645821

[16] Choy LYL Lo YMD . The longer, the better? The past, present and the future of long cell‐free DNA‐based liquid biopsy. Med Bull. (2025) 1:86–91. doi: 10.1002/mdb2.70004 41531421

