# Supplementary material for: Rezvilutamide combined with ADT in an HIV-positive patient with high-volume mHSPC on efavirenz-based antiretroviral therapy: a case report
Source: Front Oncol. 2026 Aug 10;16:1864715. doi: 10.3389/fonc.2026.1864715 (PMC13500321; doi:10.3389/fonc.2026.1864715)
Supplement: Supplementary file 1 [file Table1.docx]

**Supplementary Material S1. Literature Search Strategy**

A systematic literature search was independently conducted by the corresponding author (H.W.) on 5 July 2026 across three major databases: PubMed (via NCBI), Embase (via Elsevier), and Web of Science Core Collection (via Clarivate). The search strategy was designed to identify any published reports of rezvilutamide use in patients with prostate cancer and concurrent HIV infection receiving efavirenz-based antiretroviral therapy. No language or date restrictions were applied.

**Database 1: PubMed (National Center for Biotechnology Information)**

| **#** | **Search Query** | **Results** |
| --- | --- | --- |
| #1 | "rezvilutamide"[Supplementary Concept] OR "rezvilutamide"[All Fields] OR "SHR3680"[All Fields] | 33 |
| #2 | "HIV"[MeSH Terms] OR "HIV"[All Fields] OR "HIV infections"[MeSH Terms] OR "HIV-positive"[All Fields] | 500,456 |
| #3 | "prostatic neoplasms"[MeSH Terms] OR "prostate cancer"[All Fields] OR "prostatic cancer"[All Fields] OR "mHSPC"[All Fields] OR "metastatic hormone-sensitive prostate cancer"[All Fields] | 216,894 |
| #4 | "efavirenz"[MeSH Terms] OR "efavirenz"[All Fields] OR "CYP3A4 inducer"[All Fields] OR "cytochrome P-450 CYP3A4 inducer"[All Fields] | 6,003 |
| **#5** | **#1 AND #2 AND #3** | **0 results** |
| **#6** | **#1 AND #2 AND #3 AND #4** | **0 results** |

**Database 2: Embase (Elsevier)**

| **#** | **Search Query** | **Results** |
| --- | --- | --- |
| #1 | 'rezvilutamide'/exp OR 'rezvilutamide' OR 'shr3680' | 144 |
| #2 | 'human immunodeficiency virus'/exp OR 'HIV' OR 'HIV infection'/exp OR 'HIV positive' | 1,134,007 |
| #3 | 'prostate cancer'/exp OR 'prostate neoplasm' OR 'prostatic cancer' OR 'metastatic hormone sensitive prostate cancer' OR 'mHSPC' | 328,725 |
| #4 | 'efavirenz'/exp OR 'efavirenz' OR 'CYP3A4 inducer' | 27,102 |
| #5 | #1 AND #2 AND #3 [1 result retrieved; deemed irrelevant after full-text screening — a network pharmacology study not involving rezvilutamide, HIV comorbidity, or clinical treatment] | 1 *(irrelevant)* |
| **#6** | **#1 AND #2 AND #3 AND #4** | **0 results** |

**Database 3: Web of Science Core Collection (Clarivate)**

| **#** | **Search Query** | **Results** |
| --- | --- | --- |
| #1 | TS=("rezvilutamide" OR "SHR3680") | 52 |
| #2 | TS=("HIV" OR "HIV-positive" OR "HIV infection" OR "human immunodeficiency virus") | 507,869 |
| #3 | TS=("prostate cancer" OR "prostatic neoplasms" OR "mHSPC" OR "metastatic hormone-sensitive prostate cancer") | 276,987 |
| #4 | TS=("efavirenz" OR "CYP3A4 inducer" OR "cytochrome P450 3A4 inducer") | 7,694 |
| **#5** | **#1 AND #2 AND #3** | **0 results** |
| **#6** | **#1 AND #2 AND #3 AND #4** | **0 results** |

Conclusion

No prior clinical reports of rezvilutamide use in HIV-positive prostate cancer patients receiving efavirenz-based antiretroviral therapy were identified across any of the three databases. The single record retrieved from Embase (#5) was a non-clinical network pharmacology study that did not involve rezvilutamide treatment, HIV comorbidity, or clinical management, and was therefore excluded.
